# Dual function of a bacterial protein as an adhesin and extracellular effector of host GTPase signaling

**DOI:** 10.1080/21541248.2015.1028609

**Published:** 2015-07-09

**Authors:** Daniel Henry Stones, Anne Marie Krachler

**Affiliations:** Institute of Microbiology and Infection; School of Biosciences; University of Birmingham; Edgbaston, Birmingham, United Kingdom

**Keywords:** actin dynamics, adhesin, effector, host-pathogen interaction, lipid signaling, phosphatidic acid, Rho GTPases, RhoA, *Vibrio*

## Abstract

Bacterial pathogens often target conserved cellular mechanisms within their hosts to rewire signaling pathways and facilitate infection. Rho GTPases are important nodes within eukaryotic signaling networks and thus constitute a common target of pathogen-mediated manipulation. A diverse array of microbial mechanisms exists to interfere with Rho GTPase signaling. While targeting of GTPases by secreted bacterial effectors is a well-known strategy bacterial pathogens employ to interfere with the host, we have recently described pathogen adhesion as a novel extracellular stimulus that hijacks host GTPase signaling. The Multivalent Adhesion Molecule MAM7 from *Vibrio parahaemolyticus* directly binds host cell membrane lipids. The ensuing coalescence of phosphatidic acid ligands in the host membrane leads to downstream activation of RhoA and actin rearrangements. Herein, we discuss mechanistic models of lipid-mediated Rho activation and the implications from the infected host's and the pathogen's perspective.

*Vibrio parahaemolyticus* is an emerging pathogen and since its discovery in the 1950s it has lead to a globally disseminated pandemic of gastroenteritis.^1^ *V. parahaemolyticus* preferentially colonizes the small intestine, and food-borne infection typically manifests as watery or bloody diarrhea, nausea and vomiting. Although the disease is usually self-limiting in otherwise healthy individuals, *V. parahaemolyticus* infection can rapidly disseminate and lead to fatal septicemia in immunocompromised patients.^2^

*V. parahaemolyticus* possesses an arsenal of virulence factors, including adhesins, toxins and 2 type 3 secretion systems, which together give the pathogen the capacity to penetrate the mucosa, invade deeper tissues and disseminate to the blood stream. Although this property is usually kept in check by the host's immune system, it is important to understand the basis for the organism's invasiveness. Although invasion has been observed both in human and animal hosts, the factors facilitating this property have long remained elusive.^3,4^ It has been ruled out that the secreted toxins associated with clinical isolates, TDH and TRH, are responsible for intestinal permeability, although both contribute to enterotoxicity.^5^ Two type 3 secretion systems (T3SS) have been identified in *V. parahaemolyticus*, each carrying its own dedicated set of effectors, which are translocated into the eukaryotic cytoplasm during infection to manipulate the host to the pathogen's advantage. Although many of the effectors still remain elusive, the first T3SS, encoded by chromosome I, seems to mediate cytotoxicity, while the second T3SS, encoded by chromosome II, is required for enterotoxicity and establishment of persistence through a small intracellular niche population.^6^ However, earlier work suggests that invasiveness cannot be attributed to either T3SS1 or T3SS2 alone. T3SS1 has no effect on permeability, while T3SS2 contributed but is dispensable for bacterial transmigration.^7^

Recently, a new class of bacterial adhesins, termed Multivalent Adhesion Molecules (MAMs) was discovered and its founding member was identified in *V. parahaemolyticus*. The *V. parahaemolyticus* MAM, MAM7, is constitutively expressed and confers on bacteria the ability to attach to a wide range of different host cell types, including epithelial cells, fibroblasts and macrophages.^8^ Attachment is mediated by 2 host surface molecules: While fibronectin acts as a co-receptor to increase the rate of binding, high affinity interactions between pathogen and host surface is mediated by a group of lipid ligands, phosphatidic acids (PAs). MAM7 comprises 7 tandem mammalian cell entry (MCE) domains, each of which is capable of binding phosphatidic acid ligands, albeit with varying affinity.^9^ It has been established that targeting MAM-mediated adhesion can attenuate bacterial infection of a wide range of pathogens, including *V. parahaemolyticus*.^10^ For this purpose, we have developed a synthetic adhesion inhibitor, comprising a recombinant fragment of MAM7 chemically coupled to a polymer scaffold. Such bacteriomimetic inhibitors show vastly improved efficacy over soluble MAM-based adhesion inhibitors, by exploiting the binding avidity gained by bacterial surface display, while avoiding side effects caused by other bacterial surface components.^11^ While these adhesin-coupled beads where originally produced with therapeutic applications in mind, they have more recently lead to a serendipitous discovery regarding the interplay between adhesion and host cellular signaling. Studies using this minimalistic approach to characterize the effect of adhesion independent of other microbial factors have revealed that MAM7s ability to bind tightly to host phosphatidic acids directly activates host RhoA signaling and turns it to the pathogen's advantage.^12^

Rho GTPases constitute important nodes of eukaryotic cell signaling, at which many cellular processes, including cytoskeletal dynamics, trafficking, and proliferation intersect. As such, it is perhaps unsurprising that many microbial pathogens possess effectors which are capable of influencing Rho GTPase signaling, either by direct biochemical modification of GTPases, or by manipulation of endogenous host downstream effectors.^13^ Indeed, *V. parahaemolyticus* contains at least 2 effectors targeting Rho GTPases. VopS, a T3SS1 effector, AMPylates Rho GTPases, leading to multifaceted effects such as immune evasion and cytoskeletal collapse at later stages of infection.^14,15^ VopC, a T3SS2 effector, selectively deamidates the Rho GTPases Rac and Cdc42, but not RhoA, and is implicated in the establishment of an intracellular niche.^16^ MAM is distinct from these in several ways. Although it leads to GTPase activation, and can thus be described as a GTPase effector, it is not secreted but is a surface exposed, outer membrane-anchored bacterial protein. Also, its function is not directly conveyed by an enzymatic activity (as is the case for both T3SS effectors), but is an indirect consequence of its binding to PA, a lipid second messenger. Despite its activity being indirect, it is highly specific and is only directed at RhoA, but not Rac or Cdc42.^12^

Phosphatidic acids are phospholipids consisting of a glycerol backbone linked to a phosphate headgroup via C3 and 2 fatty acid chains via C1 and C2. Although PAs are usually turned over quickly and as such are short-lived and constitute only a minor fraction of a cell's membrane lipid composition (1–4% of total phospholipid, on average, are PAs), they are a key second messenger and a component of multiple cellular signaling pathways. PAs are involved in regulation of cellular lipid metabolism, proliferation and trafficking, among others.^17-19^ However, because of their fast-lived nature, our knowledge concerning the details of PA biochemistry, including their prevalence and distribution within different tissues, is still sparse. Thus far, studies on PAs have focused on pathways involving PA localized in the inner leaflet of the plasma membrane and cellular organelles, such as the Golgi.^20^ Although it has been shown that PA is also found in the outer leaflet of the plasma membrane, it is not characterized how this pool is generated or how it is linked to cellular functions, especially in the context of the intestinal epithelium.^21^ Characterization of the interaction between bacterial Multivalent Adhesion Molecules (MAMs) and the extracellular PA pool and of the resulting host cellular phenotypes will help to shed more light on this important group of lipid second messengers.

How exactly PA binding and clustering by MAMs leads to RhoA activation is still unknown, but several possibilities exist. Due to their negatively charged headgroups and charge repulsion, localized enrichment of PAs in the membrane induces a negative curvature in the lipid bilayer.^22^ This may lead to the recruitment of adapter proteins, which form the basis of signaling platforms that are capable of RhoA activation.^23,24^ Alternatively, sequestration of PA, which is usually subject to rapid turnover, could act as a signal – the inhibition of PA degradation to diacylglycerol or inhibited flux of PA metabolites may stimulate enzymes involved in PA synthesis and turnover, as was demonstrated for other cell types.^25,26^ Further work, capitalizing on MAM-coupled biomimetic beads as a tool to trap and characterize MAM- and PA associated signaling platforms by proteomics and lipidomics approaches, will help to shed light on the mechanism of signal transduction between PA and RhoA. While the exact mechanism linking lipid binding and RhoA activation is still unknown, it is clear that MAM7s ability to cluster phosphatidic acids in the membrane is crucial for its function. While individual MCE domains coupled to a scaffold can still bind phosphatidic acids with sufficient affinity to mediate cellular attachment, this is not sufficient to elicit GTPase signaling. Additionally, even all 7, intact tandem MCE domains are not able to activate RhoA, unless coupled to a surface.^12^ This requirement of MCE domains to be coupled to a surface, be it bacterial or polymer bead, in order to activate RhoA signaling, may indicate the need for multiple MAM molecules to be maintained in close proximity to one another to facilitate PA clustering and GTPase activation. Similarly, clustering of protein-receptor interactions on the membrane plays a key role in multiple cell signaling pathways.^27,28^

The signaling events triggered by MAM downstream of RhoA activation are better defined, although some questions still remain to be answered: RhoA activation leads to activation of LIM kinase (LIMK), which in turn results in phosphorylation of cofilin. P-cofilin inhibits actin depolymerization, which in isolated epithelial cells leads to stress fiber formation. In the canonical pathway, RhoA/ROCK signaling also leads to myosin activation, which contributes to tight junction disruption^29^, however if this is also the case if the pathway is triggered by MAM adhesion as an extracellular stimulus remains to be investigated. In context of a polarized epithelial cell layer, activation of the RhoA/LIMK/cofilin signaling axis leads to redistribution of tight junction proteins and compromised barrier function. The effect of this is 2-fold: Bacteria can migrate across the epithelial layer through paracellular movement and reach deeper tissues. Second, break-down of cell junctions depolarizes the barrier, meaning specific properties and markers of apical and basolateral surfaces are lost. We and others have shown that *V. parahaemolyticus* only binds to and infects intestinal epithelium from the apical (luminal) side.^4,12^ This asymmetry is lost through the process of depolarization. Thus, depolarization and cellular junction opening increases the surface area accessible to *V. parahaemolyticus* and speeds up bacterial attachment and rate of effector delivery (**Fig. 1**). We have shown this on the example of the T3SS1 effector VopS. During infection of polarized epithelium with wild type *V. parahaemolyticus*, VopS efficiently translocates into host cells and inactivates RhoA, leading to cell rounding, a rapid loss in transepithelial resistance, and ultimately cell death.^14^ Cells infected with a VopS deletion strain, in contrast, show strong RhoA activation, brought on by the action of MAM7. Infection with a VopS positive but MAM deleted strain, leads to intermediate RhoA activation, presumably reflecting the cell's endogenous level of active RhoA. Thus, even though VopS acts as a potent and irreversible inhibitor of RhoA, its rapid translocation and action on host cells requires MAM-mediated enhancement of RhoA activation early during infection.

**Figure 1.**
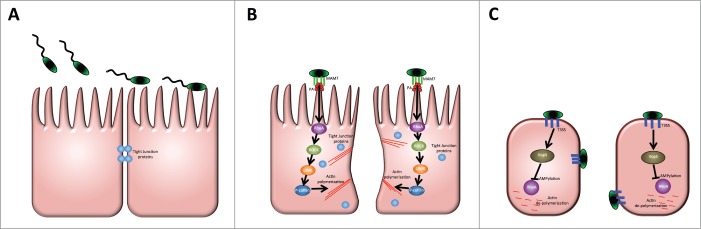
Multivalent Adhesion Molecule 7 compromises epithelial barrier integrity and accelerates T3SS effector-mediated tissue damage during infection. (**A**) Flagella-driven, highly motile *V. parahaemolyticus* (green) reach the gut epithelium and attach to the cell surface. At this point, epithelial barrier function is maintained by means of cellular junction complexes (blue). (**B**) MAM7 (green) binding to PA (red) on the epithelial surface leads to RhoA activation (purple). RhoA activation is signaled via LIMK (orange) and cofilin (blue), which results in actin rearrangements (red) and redistribution of tight junction proteins (light blue), cumulating in enhanced transepithelial permeability. (**C**) These MAM7-mediated events increase the surface area accessible to *V. parahaemolyticus*, thus increasing the efficacy of T3SS (dark blue) effector transfer. The T3SS effector VopS inhibits RhoA, thus leading to actin destabilization and cell rounding. The synergistic activities of MAM7 and T3SS effectors leads to bacterial transmigration across the epithelial barrier.

Similar sequences of temporal activation and deactivation of cellular activities by effectors are a conserved theme in bacterial pathogenesis. *Legionella pneumophila*, for example, sequentially activates and later deactivates the small GTPase Rab1 during infection, to direct modification of its intracellular niche.^30,31^ These examples of the dynamic interplay between 2 seemingly opposing activities demonstrate the necessity of tight coordination between different pathogen-derived cues to achieve successful infection.
